# Survival predictors associated with signet ring cell carcinoma of the esophagus (SRCCE): A population-based retrospective cohort study

**DOI:** 10.1371/journal.pone.0181845

**Published:** 2017-07-26

**Authors:** Zihao Wan, Zhihao Huang, Liaobin Chen

**Affiliations:** 1 Department of Orthopedic Surgery, Zhongnan Hospital of Wuhan University, Wuhan, Hubei Province, China; 2 Department of Colorectal and Anal Surgery, Zhongnan Hospital of Wuhan University, Wuhan, Hubei Province, China; National Cancer Center, JAPAN

## Abstract

**Purpose:**

Signet ring cell carcinoma of the esophagus (SRCCE) is an uncommon tumor associated with significant morbidity and mortality. There is still no consensus regarding cut-off values for tumor size, age and optimal treatment for SRCCE. Thus, we elucidated the current survival outcomes of patients with SRCCE and analyzed factors associated with prognosis.

**Methods:**

A retrospective cohort study based on the SEER (The Surveillance, Epidemiology, and End Results) program database was conducted. We identified 537 patients (461 men and 76 women) newly diagnosed with SRCCE between January 2004 and December 2014. A multivariate Cox proportional hazards model was utilized to measure the mortality-associated risk factors in patients with SRCCE after adjusting for various variables.

**Results:**

The 1-, 2- and 5-year disease-specific mortalities (DSM) were 51.6%, 67.6%, and 78.4%, respectively, and the median survival time was 12.0 months. The factors correlated with mortality hazard were marital status (unmarried versus married, Hazard Ratio (HR) = 1.443), tumor size (≥ 5 cm versus < 5 cm, HR = 1.444), tumor grade (high grade versus low grade, HR = 3.001), condition of primary tumor (T4 versus T1, HR = 2.178), regional lymph node metastasis (N1 versus N0, HR = 1.739), further metastasis (M1 versus M0, HR = 1.951) and chemotherapy (receiving chemotherapy versus no chemotherapy, HR = 0.464).

**Conclusions:**

The contemporary 5-year DSM was 78.4%. Being unmarried, having a tumor size ≥ 5 cm, a high tumor grade, a score of T4 for tumor invasion of adjacent organs, a score of N1 for regional lymph node metastasis, a score of M1 for distant metastasis and no chemotherapy were independent predictors of high DSM.

## Introduction

Signet ring cell carcinoma is a particular pathological type of carcinoma that contains mucilage pushing the nucleus to the periphery, causing the cancer cell to resemble a signet-ring [1–3]. It has been estimated that 3.5–5.0% of all esophageal cancers are SRCCE [4–6]. The World Health Organization has classified SRC as a particular kind of adenocarcinoma [7]. Several studies have indicated that this kind of aggressive tumor is generated from a cancer stem cell and is associated with poor prognosis [6, 8, 9]. While a series of studies regarding signet ring cell carcinoma (SRCC) have already been carried out concerning gastric and colorectal cancer, our knowledge of the pathogenesis and prognostic implication of SRCCE is quite limited, and no consensus has been reached regarding its biological behavior. Previous studies of SRCC in gastric cancer have found that SRCC occurs more frequently in women and younger patients [10, 11]. In Asian countries, the incidence of gastric SRCC has been significantly increasing [12–15].

To our knowledge, the clinical management of SRCCE is subject to debate, and no randomized controlled trials have been performed to identify optimal therapeutic strategies. In the modern era, surgical resection with preoperative chemoradiation is the main approach used for the treatment of localized tumors [16–20]. Nevertheless, data analysis concerning SRCCE survival and related prognostic elements based on nationwide population studies is inadequate. The objective of this study was to employ the SEER database to demonstrate the survival conditions and distinguish independent factors associated with predicting prognosis in patients with SRCCE.

The Surveillance, Epidemiology, and End Results (SEER) Program [21] is backed by the National Cancer Institute and has provided information on tumor statistics since 1973. It gathers data on cancer cases diagnosed throughout the United States, with an estimated 28% of the US population covered. The SEER registry is a validated database that is frequently utilized in studies on cancer survival. Because it is a de-identified public-use database, the National Cancer Institute does not require institutional review board approval for SEER studies.

## Methods

### Data sources

**Fig 1:**
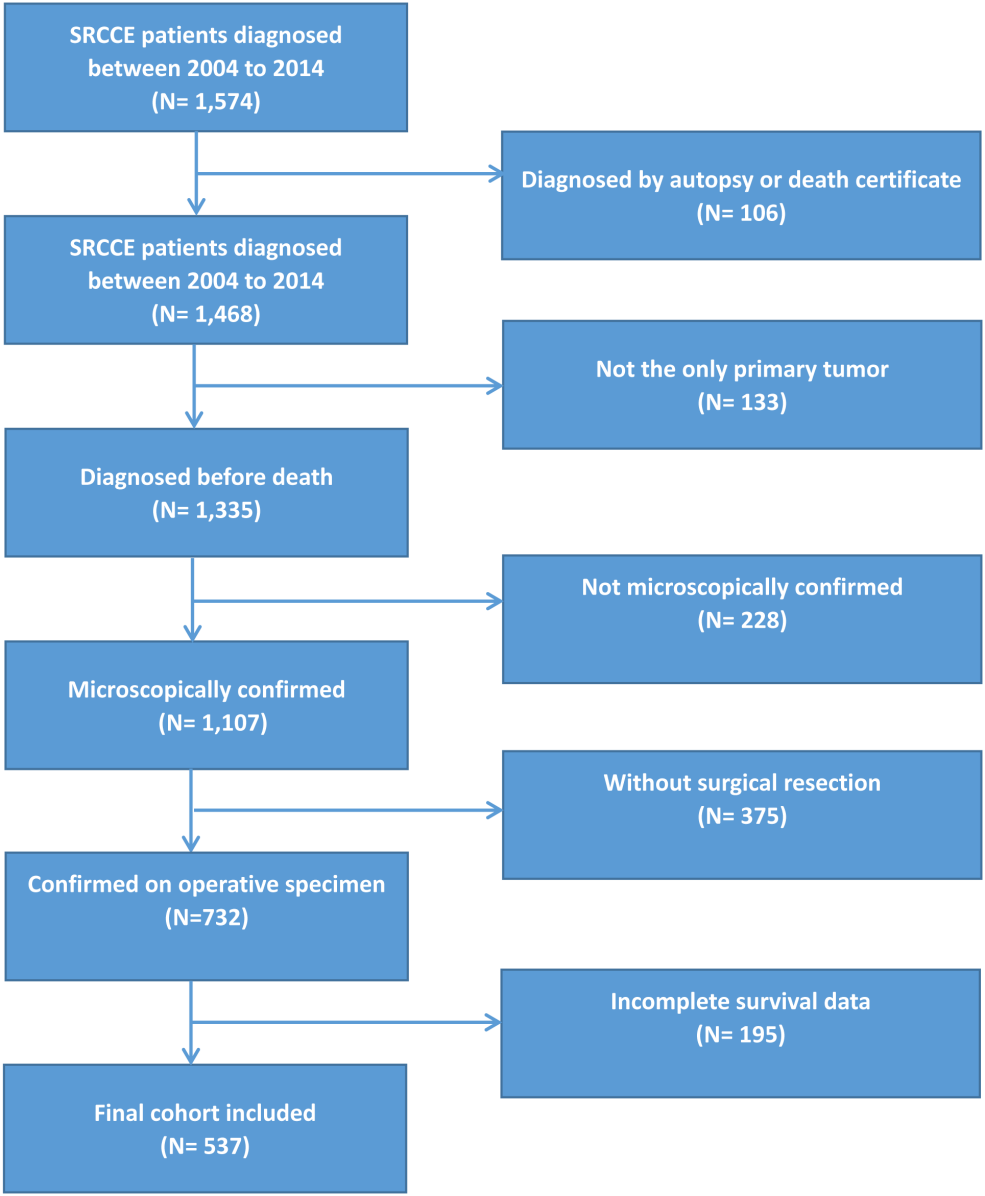
Flow chart for selection of the study cohort.

SRCCE data extracted from the SEER database (Incidence-SEER 18 Regs Custom Data (with additional treatment fields), Nov 2016 Sub (1973–2014 varying)) were employed to perform this population-based study from January 2004 to December 2014. Histologic International Classification of Diseases (ICD) codes, third version (ICD-0-3) were used to identify signet ring cell carcinoma. Site specific codes (C15.0-C15.5, C15.8, C15.9) were used to screen for tumors originating in the esophagus. The following primary data were drawn from the database for analysis: year of diagnosis, age at diagnosis, sex, marital status, race, tumor site, tumor size, tumor grade, extension of primary tumor, regional lymph node metastasis, distant metastasis, treatment modality, cause of death, and survival time. Cases without survival status and survival time were excluded. Patients diagnosed by either autopsy or death certificate were excluded. Those who had secondary malignancies at the time of diagnosis as well as patients who did not undergo surgical resection or were not confirmed with operative specimens were also excluded. The inclusion and exclusion procedure is shown in a flow chart (Fig 1). Well-differentiated and moderately differentiated histologic features were defined as low grade, while poorly differentiated and undifferentiated histologic types were classified as high grade. pTNM was used in patients undergoing tumor resection because they were microscopically confirmed. The American Joint Commission on Cancer staging system 6th edition has been employed in the SEER database since 2004. The data from 2014 through 2017 were the latest in this database at the beginning of this study, so we choose the period (2004 to 2014) for our study.

### Statistical analysis

The basic outcome of the investigation was DSM. Descriptive statistics were computed for all variables. Multi-collinear diagnostic analysis was conducted, and variance inflation factors less than 10 meant that there was no co-linearity between the variables. We employed multiple-imputation using Monte Carlo Markov Chain methods to estimate missing values. The Cox proportional hazards model was used to conduct univariate and multivariate analysis and calculate the mortality-associated risk factors in those with SRCCE after adjusting for a series of indexes. We calculated hazard ratios (HRs) and the 95% confidence interval (CI) by means of the Cox proportional hazards model to assess the HR of mortality in patients with SRCCE. The Kaplan-Meier method was carried out to generate survival curves. SEER*Stat version 8.3.4 (IMS Inc. USA) was used to search for relevant cases. SPSS version 23 (IBM Corp, USA) was used for statistical analysis. The R statistical software, version 3.3.1 (www.r-project.org) was employed to perform multiple-imputation. Deviations between groups were considered statistically significant at P < 0.05.

### Ethical statements

The National Cancer Institute and the Ethics Review Board of Zhongnan Hospital of Wuhan University considered institutional review board approval to be unnecessary because this SEER study is based on a de-identified public-use database.

## Results

During the 11-year study period, we included 537 patients with signet ring cell carcinoma of the esophagus (461 men and 76 women). Table 1 illustrates the distribution of patients’ characteristics in the investigation. Men accounted for 85.84% of patients. The mean (SD) age at diagnosis was 67.2 (12.3) years. The ages ranged from 23 to 100 years. Approximately 60.9% of the included patients were married, and 94.1% were Caucasian. More than 85% of SRCCE cases originated from the lower third of the esophagus. In all, 95.6% of tumors were histologically confirmed to be poorly differentiated or undifferentiated tumors of high grade. Tumors invading adjacent organs accounted for 16.4% of samples. Over 50% had SRCCE metastasizing to regional lymph nodes, and tumors metastasizing to distant locations made up 35.8% of the sample. The mean and median tumor size at the time of diagnosis was 5.6 (3.9) and 5.0 cm, respectively. A total of 180 patients (33.6%) underwent surgery alone. A total of 119 (22.1%) patients received radiotherapy after surgery, while less than 44.3% underwent radiotherapy prior to surgery and more than 60% of patients underwent chemotherapy. From 2010 to 2014, 255 patients were included, and SRCCE metastasizing to bone, liver, lung and brain accounted for 9.4%, 8.6%, 5.1% and 0.4% of patients, respectively.

**Table 1:** Characteristics of patients with signet ring cell carcinoma of the esophagus.

Characteristic	Total		Male		Female	
	NO.	%	NO.	%	NO.	%
**Patients**	537	100	461	100	76	100
**Age, year**						
Mean(SD)	67.2 (12.3)		66.8 (11.8)		69.4 (14.5)	
Median	67.0		67.0		70.0	
**Marital status**						
Married	327	60.9	300	64.9	27	35.5
Unmarried ^a^	179	33.4	134	29.2	45	59.2
Unknown	31	5.7	27	5.9	4	5.3
**Race**						
Caucasian	505	94.1	438	95.1	67	88.2
Afro-American	16	2.9	10	2.2	6	7.9
Other ^b^	14	2.7	11	2.3	3	3.9
Unknown	2	0.3	2	0.4	0	0
**Tumor site**						
Upper third of esophagus	5	0.9	3	0.7	2	2.6
Middle third of esophagus	39	7.3	32	6.9	7	9.2
Lower third of esophagus	471	87.8	406	88.0	65	85.5
Overlapping lesion of esophagus	22	4.0	20	4.4	2	2.6
**Tumor grade**						
Low	24	4.4	19	4.2	5	6.6
High	513	95.6	442	95.8	71	93.4
**Primary tumor**						
T1	171	31.9	139	30.2	32	42.1
T2	49	9.1	43	9.4	6	7.9
T3	229	42.6	202	43.8	27	35.5
T4	88	16.4	77	16.6	11	14.5
**Regional lymph node**						
N0	234	43.6	198	42.9	36	47.4
N1	303	56.4	263	57.1	40	52.6
**Metastasis**						
M0	345	64.2	297	64.5	48	63.2
M1	192	35.8	164	35.5	28	36.8
**Surgery and radiotherapy**						
Surgery alone	180	33.6	144	31.2	36	47.4
Radiotherapy prior to surgery	238	44.3	212	45.9	26	34.2
Radiotherapy after surgery	119	22.1	105	22.9	14	18.4
**Chemotherapy**						
Receiving chemotherapy	336	62.6	300	65.0	36	47.4
No chemotherapy	201	37.4	161	35.0	40	52.6
**Tumor size, cm**						
Mean(SD)	5.6 (3.9)		5.8 (4.0)		4.5 (2.5)	
Median	5.0		5.0		4.0	
**Distant metastases from 2010 to 2014**						
	255	100	190	100	65	100
**Bone metastasis**						
Yes	24	9.4	19	10.0	5	7.7
No	231	90.6	171	90.0	60	92.3
**Liver metastasis**						
Yes	22	8.6	16	8.4	6	9.2
No	233	91.4	174	91.6	59	90.8
**Brain metastasis**						
Yes	1	0.4	1	0.5	0	0
No	254	99.6	189	99.5	65	100
**Lung metastasis**						
Yes	13	5.1	9	4.7	4	6.2
No	242	94.9	181	95.3	61	93.8

^**a**^ Including separated, divorced, widowed and single;

^**b**^ Including Asian/Pacific Islander and American Indian/Alaskan Native.

Table 2 demonstrates the results of univariate and multivariate Cox proportional hazards analyses for the mortality-associated risk factors in patients with SRCCE. Unmarried status (HR = 1.443, 95% CI (1.102, 1.890)), tumor size ≥ 5 cm (HR = 1.444, 95% CI (1.096, 1.904)), high tumor grade (HR = 3.001, 95% CI (1.107, 8.130)), T4 (HR = 2.178, 95% CI (1.453, 3.265)), N1 (HR = 1.739, 95% CI (1.296, 2.333)), and M1 (HR = 1.951, 95% CI (1.455, 2.614)) were associated with an increased risk of mortality, while chemotherapy (HR = 0.464, 95% CI (0.327, 0.659)) was a protective factor for survival. However, no clear difference was noted for the prognosis with surgery alone, radiotherapy prior to surgery or radiotherapy after surgery in the multivariate analysis.

**Table 2:** Cox model with hazard ratios and 95% confidence intervals of mortality associated with covariates in patients with signet ring cell carcinoma of the esophagus.

Variable	Crude			Adjusted ^a^		
	HR	(95% CI)	P Value	HR	(95% CI)	P Value
**Age, year**	0.994	(0.988, 1.001)	0.121	/	/	/
**Sex**						
Female	1	(reference)		/	/	/
Male	0.875	(0.707, 1.084)	0.222	/	/	/
**Marital status**						
Married	1	(reference)		1	(reference)	
Unmarried ^b^	1.387	(1.143, 1.682)	< 0.001	1.443	(1.102, 1.890)	0.008
**Race**						
Caucasian	1	(reference)		/	/	/
Afro-American	1.025	(0.663, 1.583)	0.912	/	/	/
Other ^c^	0.921	(0.576, 1.471)	0.731	/	/	/
**Tumor site**						
Upper third of esophagus	1	(reference)		/	/	/
Middle third of esophagus	0.908	(0.359, 2.297)	0.839	/	/	/
Lower third of esophagus	1.037	(0.430, 2.505)	0.935	/	/	/
Overlapping lesion of esophagus	1.748	(0.683, 4.476)	0.244	/	/	/
**Tumor size, cm**						
< 5.0	1	(reference)		1	(reference)	
≥ 5.0	1.855	(1.502, 2.290)	< 0.001	1.444	(1.096, 1.904)	0.009
**Tumor grade**						
Low	1	(reference)		1	(reference)	
High	2.011	(1.183, 3.418)	0.010	3.001	(1.107, 8.130)	0.031
**Primary tumor**						
T1	1	(reference)		1	(reference)	
T2	0.643	(0.450, 0.920)	0.016	1.220	(0.724, 2.055)	0.455
T3	0.894	(0.730, 1.095)	0.278	1.221	(0.868, 1.717)	0.251
T4	2.034	(1.606, 2.576)	< 0.001	2.178	(1.453, 3.265)	< 0.001
**Regional lymph node**						
N0	1	(reference)		1	(reference)	
N1	1.446	(1.226, 1.706)	< 0.001	1.739	(1.296, 2.333)	< 0.001
**Metastasis**						
M0	1	(reference)		1	(reference)	
M1	2.911	(2.475, 3.423)	< 0.001	1.951	(1.455, 2.614)	< 0.001
**Surgery and radiotherapy**						
Surgery alone	1	(reference)		1	(reference)	
Radiotherapy prior to surgery	1.016	(0.712, 1.450)	0.930	0.794	(0.430, 1.468)	0.462
Radiotherapy after surgery	1.726	(1.080, 2.759)	0.022	1.265	(0.668, 2.395)	0.470
**Chemotherapy**						
No chemotherapy	1	(reference)		1	(reference)	
Receiving chemotherapy	0.552	(0.472, 0.644)	< 0.001	0.464	(0.327, 0.659)	< 0.001

^**a**^ Adjusted for marital status, tumor size, tumor grade, primary tumor, regional lymph node, metastasis and treatment;

^**b**^ Including separated, divorced, widowed and single;

^**c**^ Including Asian /Pacific Islander and American Indian/Alaskan Native.

**Fig 2:**
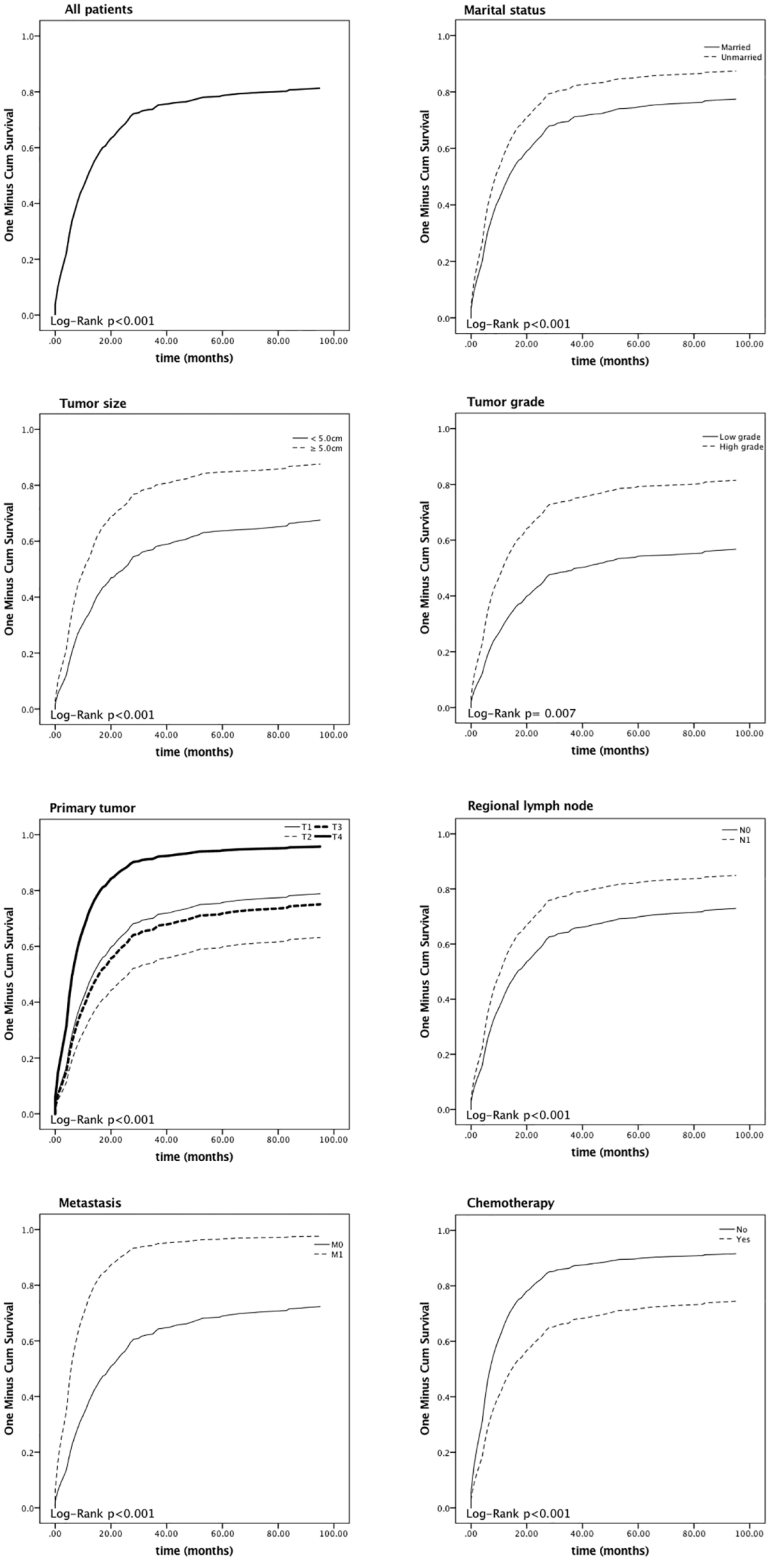
Kaplan—Meier survival curves of all patients and patients stratified by various factors.

**Fig 3:**
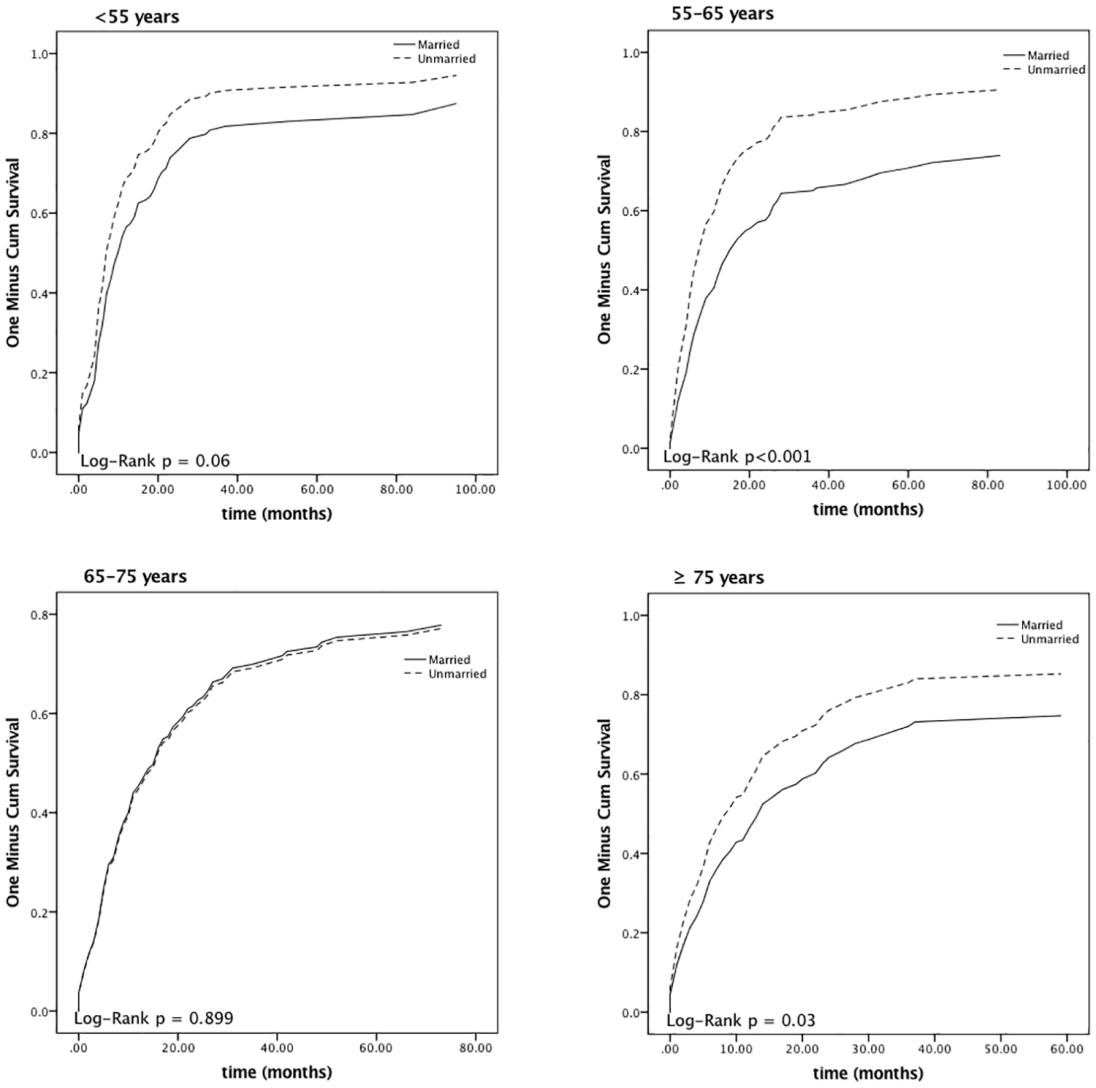
Kaplan—Meier survival curves for patients with different marital status stratified by various age groups.

Fig 2 shows the survival curves for all individuals included and patients stratified by different independent predictors. The 1-, 2- and 5-year disease-specific mortalities (DSM) were 51.6%, 67.6%, and 78.4%, respectively. The median survival time was 12.0 months. The 5-year relative death rate was 84.8% higher in patients who were unmarried; Fig 3 shows the survival curves of patients of different marital status stratified by various age groups. Patients with tumor sizes < 5 cm had a lower 5-year relative excess risk of mortality than those with a tumor size ≥ 5 cm (61.7% versus 86.8%; P < 0.001). Positive associations were also observed between tumor development and mortality. The 5-year DSMs were 96.4%, 84.3%, and 95.5% for SRCCE-invading adjacent organs, with encroachment on regional lymph node follicles and metastasis to distant locations, respectively. Furthermore, the 5-year DSM for those receiving chemotherapy was 76.9%, which was lower than the 80.4% observed in patients who did not undergo chemotherapy.

## Discussion

SRCCE is an extremely rare oncologic abnormality that may originate from cancer stem cells, with the propensity to metastasize to distant sites at an early stage; to date, limited research on SRCCE survival has been reported. This cancer typically affects young women and has a poor prognosis [8, 9]. The correct management of these tumors is controversial and is probably affected by tumor size, infiltration and dissemination [16–19]. Therefore, we employed the SEER database to identify the survival rate and mortality-related factors over an 11-year period (from January 2004 to December 2014). In our study, the 1-, 2-, 3- and 5-year disease-specific mortalities (DSM) were 51.6%, 67.6%, 74.1% and 78.4% (in other words, 1-, 2-, 3- and 5-year disease-specific survival rates (DSS) were 48.4%, 32.4%, 25.9% and 21.6%), respectively. This calculation might be ambiguous, and here we provide the conversion formula. The median survival time was 12.0 months, although these outcomes were reported to be poorer in cases of advanced gastric signet ring cell carcinoma studied in a prior investigations [22]. Lei et al. [23] observed that the 3- and 5-year survival rates were 37.6% and 25.9%, respectively. Nafteux et al. [5] reported that the 5-year DSS was 22.4%. Enlow et al. [6] reported that the 3-year survival was 27.3%. Our survival results are relatively consistent with these studies.

DSM=1−DSS

In our 537 participants, men and women accounted for 85.8% and 14.2% of cases, respectively. The mean age for our patients was 67.2 years. In Enlow’s [6] study, men and women made up 91.0% and 9.0%, respectively, and the mean age was 66 years. Philippe and Nafteux et al. [5] reported that male and female patients accounted for 83.3% and 16.7%, respectively, and the mean age was 64.1 years. In a study of 596 SRCCE patients, Sai and Miriam et al. [4] stated that the proportion of men and women were 85.1% and 14.9, respectively, and the mean age was 66.9 years. These outcomes were in accordance with our findings. Thus, we concluded that SRCCE usually occurrs in older male individuals and has a poor prognosis.

The influence of age and sex on survival has always been a topic of debate, with adverse outcomes reported by different studies. In our study, the included patients with SRCCE were relatively old, with a median age of 67.2 years at diagnosis and were predominantly male, which is inconsistent with previous reports in the international literature concerning gastric signet ring cell carcinoma, which showed that SRCC is predominantly found in young women [8, 9]. In our study, age and sex were found to insignificantly affect prognosis in univariate analysis; however, Song et al. [24] reported that age and sex were independent risk factors for esophageal adenocarcinoma. The most frequent site of SRCCE delineated in the database was the lower third of the esophagus, which is consistent with previous reports [4]. However, no significant survival deviation was found for age, sex and tumor site in the present study.

Marital status was a prognostic indicator in our investigation. Unmarried patients aged from 55 to 65 years or more than 75 years were prone to have a shorter survival time than married patients. The importance of marital status has already been reported. It is considered to be an independent predictor for prognosis in various malignancies, and those who are married exhibit better survival in colorectal cancer, prostate cancer, and mammary gland cancer [25–27]. Marital status can impact coping strategies, quality of life, and emotion as a significant component of social support [28, 29]. Furthermore, Aizer et al. [25] elucidated that the survival benefit of marriage outweighs that of chemotherapy in esophageal cancer, and marital status had a greater influence in men. However, further prospective cohort investigations are required to validate any effect of marital status on survival outcomes in patients suffering from SRCCE because we are not able to establish a causal relationship between marital status and prognosis from a retrospective study.

A sharp difference in the tumor’s physical magnitude was also found. In our research, the mean size at presentation was 5.6 cm, and we noticed that a size larger than 5.0 cm was associated with a significant negative impact on survival not only in univariate analysis but also in the multivariate Cox regression model. However, no standard size cutoff that indicates an abrupt change in the prognosis of SRCCE has been agreed upon. There have been differing conclusions on the impact of tumor size on survival by different esophageal carcinoma studies. Francisco and Agoston et al. [30, 31] declared that the relationship between the size of the oncologic entity with a worse prognosis in esophageal adenocarcinoma was not proven. In a retrospective study of 273 patients at the University of Texas MD Anderson Cancer Center and the University of Rochester Medical Center, Gaur et al. [32] identified tumor size as an independent prognostic indicator and demonstrated that an endoscopically measured tumor length >2 cm predicted a decline in long-term survival.

Additionally, according to our research, more advanced SRCCE adversely affected the survival time. Patients with intrusion of the neoplasm into neighboring organs, regional lymph node metastasis and distant transfer showed much shorter survival times, with consistent conclusions found in early studies. Twine and Shoji et al. [33, 34] reported lymph node metastases as an essential predictor and emphasized the significant role of preoperative ultrasound in predicting prognosis in SRCCE patients with regional lymph node metastasis. Nevertheless, in a study of 93 patients at Brigham & Women's Hospital and Massachusetts General Hospital, Agoston et al. [31] did not find statistical significance in these factors, including tumor T, N stage and neoplasm volume, on prognosis. In our study, patients with distant metastases accounted for 35.8% of patients, and from 2010 to 2014, tumors metastasizing to liver, bone, lung and brain tissue made up 8.6%, 9.4%, 5.1% and 0.4% of cases, respectively. In previous studies on esophageal-gastric adenocarcinoma, SRC showed a higher risk of peritoneal carcinomatosis at initial diagnosis and up to a 50% recurrence rate of peritoneal carcinomatosis [35, 36]. Furthermore, comprehensive managements for the treatment of peritoneal carcinomatosis, including Cytoreductive Surgery and Hyperthermic Intraperitoneal Chemotherapy, have already been suggested and showed encouraging results [37, 38]. However, in a retrospective study of 816 cases, Akiko et al. [39] found that adenocarcinoma of the esophagogastric junction had more regional lymph node and lung metastasis but less peritoneal metastasis compared with gastric adenocarcinoma.

In our analysis, the proportion of patients undergoing surgery alone, radiotherapy prior to surgery, and radiotherapy after surgery accounted for 33.6%, 44.3%, and 22.1% of patients, and 62.6% of all patients experienced chemotherapy, all of which indicated that surgery, radiotherapy and chemotherapy were the mainstream managements of SRCCE. [40–43]. Through multivariate analysis of the whole cohort, we found that receiving chemotherapy could significantly improve survival in contrast to no chemotherapy, while no significant difference was found between surgery resection alone, radiotherapy prior to surgery, or radiotherapy after surgery, despite the fact that patients who underwent radiation after operation showed a worse prognosis in univariate analysis. Esophagectomy has been considered the preferred approach for localized SRCCE. Over the past twenty years, radiotherapy alone has been used only when the malignancy was nonresectable [40]. In a study of 400 patients, Lawrence and Mariette et al. [40, 44] reported that the use of radiation therapy combined with chemotherapy and multimodal therapeutic strategies could contribute to a superior prognosis in contrast to those achieved with radiotherapy alone. In addition, Chirieac et al. [20] found similar complete pathologic response rates in both SRCCE and non-SRCCE patients after management with induction therapy. However, Jonathon and Chadrick et al. [6] demonstrated that no complete pathologic response was detected in patients in a signet ring cell group after preoperative neoadjuvant chemoradiation compared with an over 30% response in non-signet ring cell patients. The comparative position of induction therapy remains contentious, and further investigations are warranted to clarify these contrasting outcomes.

The treatment guidelines for esophageal carcinoma have changed over time. In a study of 422 cases from the CROSS trials, Oppedijk et al. [45] indicated new adjuvant radiotherapy and chemotherapy in esophageal cancer can reduce the risk of local recurrence and peritoneal metastasis. In the MAGIC study [46], 503 patients with gastric and esophageal adenocarcinoma were randomly divided into a neoadjuvant chemotherapy group and an operation group. The surgical resection rates were 79.3% and 70.3%, respectively. Subgroup analysis suggested that neoadjuvant chemotherapy improved survival rate. Based on the MAGIC study, Western countries use neoadjuvant chemotherapy as a standard treatment modality for adenocarcinoma at the junction of the gastroesophageal junction. In the JCOG9907 study [47], a randomized controlled study comparing the survival of patients with stage II or III thoracic esophageal cancer, Japanese researchers found neoadjuvant chemotherapy offered a significant survival advantage over postoperative chemotherapy, and neoadjuvant chemotherapy has therefore been used as a standard treatment for stage II or III thoracic esophageal cancer in Japan. Most of the SRC carcinomas in the gastroesophageal junction originate from the stomach, and the management for SRC cancers originating from the esophagus (gastroesophageal junction cases are not included) can follow the above guidelines [48, 49].

Our analysis was based on the data documented in the SEER database, so we must mention a few limitations of our study. First, several variables, including comorbidities, surgical margins, the extent of resection and tumor recurrence, were missing or were not recorded in the database. Second, because of the anonymous principle of the SEER program, it was impossible for us to contact the patients in order to gain additional information. Finally, it should also be noted that, due to the existence of confounders, the results deduced from a retrospective analysis are normally of a lower methodological grade compared to those from randomized controlled trials. Despite the fact that no significant difference was found between surgery alone, radiotherapy prior to surgery and radiotherapy after surgery, receiving chemotherapy can definitively extend the survival period compared with no chemotherapy.

In sum, the SEER program database presents significant advantages, despite these restrictions, and provides the feasibility to conduct such research based on a large population with a rare malignancy. Future studies should be focused on the neoplasm’s biological behaviors and the therapeutic efficacy of SRCCE.

## Conclusions

The contemporary 5-year DSM of SRCCE is 78.4%. Social-demographic and clinical predictors, including being unmarried, having a tumor size ≥ 5 cm, a high tumor grade, tumor invasion of adjacent organs, regional lymph node metastasis, distant metastasis and no chemotherapy, are independent prognosticators for high DSM, while radiotherapy after surgery indicated worse survival in our univariate analysis model. Considering the lack of information for surgical margins, extent of resection, comorbidities and neoplasm recurrence, which affect the patients’ prognosis, further investigations combined with multiple fields are expected to elucidate favorable treatment strategies for SRCCE.

## Supporting information

S1 Checklist STROBE_checklist_v4_combined_PlosMedicine.docx. (DOCX)

## Abbreviations

CI: Confidence interval
DSM: Disease-specific mortality
HR: Hazard Ratio
ICD: International Classification of Diseases
SD: Standard deviation
SEER: The Surveillance, Epidemiology, and End Results
SRCCE: signet ring cell carcinoma of esophagus
