# Supplementary material for: Survival predictors associated with signet ring cell carcinoma of the esophagus (SRCCE): A population-based retrospective cohort study
Source: PLoS One. 2017 Jul 26;12(7):e0181845. doi: 10.1371/journal.pone.0181845 (PMC5528994; doi:10.1371/journal.pone.0181845)
Supplement: S1 Checklist — (DOCX) [file pone.0181845.s001.docx]

STROBE Statement—checklist of items that should be included in reports of observational studies

|  | Item No. | Recommendation | Page  No. | Relevant text from manuscript |
| --- | --- | --- | --- | --- |
| **Title and abstract** | 1 | (*a*) Indicate the study’s design with a commonly used term in the title or the abstract | Page 1 | Survival augurs associated with signet ring cell carcinoma of the esophagus (SRCCE): A population-based retrospective cohort study of 1046 cases. |
|  |  | (*b*) Provide in the abstract an informative and balanced summary of what was done and what was found | Page 2 | A retrospective cohort study based on SEER (The Surveillance, Epidemiology, and End Results) program database was conducted. We identified 1046 patients (896 men and 150 women) freshly diagnosed SRCCE from January 2004 to December 2013. The multivariate Cox proportional hazards model was carried out to measure the mortality-association risk factors in patients with SRCCE after adjusting for various variables. |
| Introduction | | | |  |
| Background/rationale | 2 | Explain the scientific background and rationale for the investigation being reported | Page 2-3 | Signet ring cell carcinoma is a special pathological type which is full of mucilage pushing the nucleus to the periphery and causing the appearance of the cancer cell resembles signet-ring. Some literatures indicated that this kind of aggressive tumor generates from the cancer stem cell and is associated with poor prognosis. While serious of studies regarding the signet ring cell carcinoma (SRCC) have already been carried out in gastric and colorectal cancer, our knowledge about the pathogenesis and prognostic implication of SRCCE is quite limited and no census has been reached upon the biological behavior of it. |
| Objectives | 3 | State specific objectives, including any prespecified hypotheses | Page 3 | The mainspring of this study was to employ the SEER database to demonstrate the survival upshot and distinguish independent factors associated with the prognosis in patients with SRCCE. |
| Methods | | | |  |
| Study design | 4 | Present key elements of study design early in the paper | Page 4 | Data of SRCCE extracted from the SEER database (Incidence-SEER 18 Regs Research Data + Hurricane Katrina Impacted Louisiana Cases, Nov 2015 Sub (1973-2013 varying)) were employed to execute this population-based research from January 2004 to December 2013. |
| Setting | 5 | Describe the setting, locations, and relevant dates, including periods of recruitment, exposure, follow-up, and data collection | Page 4 | Data of SRCCE extracted from the SEER database (Incidence-SEER 18 Regs Research Data + Hurricane Katrina Impacted Louisiana Cases, Nov 2015 Sub (1973-2013 varying)) were employed to execute this population-based research from January 2004 to December 2013. |
| Participants | 6 | (*a*) *Cohort study*—Give the eligibility criteria, and the sources and methods of selection of participants. Describe methods of follow-up  *Case-control study*—Give the eligibility criteria, and the sources and methods of case ascertainment and control selection. Give the rationale for the choice of cases and controls  *Cross-sectional study*—Give the eligibility criteria, and the sources and methods of selection of participants | Page 4 | Data of SRCCE extracted from the SEER database (Incidence-SEER 18 Regs Research Data + Hurricane Katrina Impacted Louisiana Cases, Nov 2015 Sub (1973-2013 varying)) were employed to execute this population-based research from January 2004 to December 2013. |
|  |  | (*b*) *Cohort study*—For matched studies, give matching criteria and number of exposed and unexposed  *Case-control study*—For matched studies, give matching criteria and the number of controls per case | Page 4-5 | Histologic International Classification of Diseases (ICD) codes, third version (ICD-0-3) were used to identify signet ring cell carcinoma (8490/3). Site specific codes (C15.0-C15.5, C15.8, C15.9) were used to screen out tumors originating in the esophagus. |
| Variables | 7 | Clearly define all outcomes, exposures, predictors, potential confounders, and effect modifiers. Give diagnostic criteria, if applicable | Page 4 | Histologic International Classification of Diseases (ICD) codes, third version (ICD-0-3) were used to identify signet ring cell carcinoma (8490/3). Site specific codes (C15.0-C15.5, C15.8, C15.9) were used to screen out tumors originating in the esophagus. The following primary data were drawn from the database for analysis: age at diagnosis, sex, marital status, race, tumor site, tumor size, tumor grade, extension of primary tumor, regional lymph node metastasis, distant metastasis, treatment modality, cause of death, and survival months. |
| Data sources/ measurement | 8* | For each variable of interest, give sources of data and details of methods of assessment (measurement). Describe comparability of assessment methods if there is more than one group | *Page 4* | The following primary data were drawn from the database for analysis: age at diagnosis, sex, marital status, race, tumor site, tumor size, tumor grade, extension of primary tumor, regional lymph node metastasis, distant metastasis, treatment modality, cause of death, and survival months. |
| Bias | 9 | Describe any efforts to address potential sources of bias | Page 4 | The Cox proportional hazards model was used to conduct univatiate and multivariate analysis and calculate the mortality-association risk factors in those with SRCCE after adjusting for a series of indexes. |
| Study size | 10 | Explain how the study size was arrived at | Page 4 | Data of SRCCE extracted from the SEER database (Incidence-SEER 18 Regs Research Data + Hurricane Katrina Impacted Louisiana Cases, Nov 2015 Sub (1973-2013 varying)) were employed to execute this population-based research from January 2004 to December 2013. |

Continued on next page

| Quantitative variables | 11 | Explain how quantitative variables were handled in the analyses. If applicable, describe which groupings were chosen and why | Page 4 | The Cox proportional hazards model was used to conduct univatiate and multivariate analysis and calculate the mortality-association risk factors in those with SRCCE after adjusting for a series of indexes. We calculated hazard ratios (HRs) and the 95% confidence interval (CI) by the means of the Cox proportional hazards model to assess the HR of mortality in patients with SRCCE. The Kaplan-Meier method was carried out to draw the survival curves. |
| --- | --- | --- | --- | --- |
| Statistical methods | 12 | (*a*) Describe all statistical methods, including those used to control for confounding | Page 4 | The Cox proportional hazards model was used to conduct univatiate and multivariate analysis and calculate the mortality-association risk factors in those with SRCCE after adjusting for a series of indexes. We calculated hazard ratios (HRs) and the 95% confidence interval (CI) by the means of the Cox proportional hazards model to assess the HR of mortality in patients with SRCCE. The Kaplan-Meier method was carried out to draw the survival curves. |
|  |  | (*b*) Describe any methods used to examine subgroups and interactions | Page 4-5 | The Cox proportional hazards model was used to conduct univatiate and multivariate analysis and calculate the mortality-association risk factors in those with SRCCE after adjusting for a series of indexes. We calculated hazard ratios (HRs) and the 95% confidence interval (CI) by the means of the Cox proportional hazards model to assess the HR of mortality in patients with SRCCE. The Kaplan-Meier method was carried out to draw the survival curves.  Deviations between groups were considered statistically significant at the P < 0.05 threshold. |
|  |  | (*c*) Explain how missing data were addressed | Page 4 | Cases without survival time and status were excluded. |
|  |  | (*d*) *Cohort study*—If applicable, explain how loss to follow-up was addressed  *Case-control study*—If applicable, explain how matching of cases and controls was addressed  *Cross-sectional study*—If applicable, describe analytical methods taking account of sampling strategy |  |  |
|  |  | (*e*) Describe any sensitivity analyses | Page 4-5 | The Cox proportional hazards model was used to conduct univatiate and multivariate analysis and calculate the mortality-association risk factors in those with SRCCE after adjusting for a series of indexes.  Deviations between groups were considered statistically significant at the P < 0.05 threshold. |
| Results | | | | |
| Participants | 13* | (a) Report numbers of individuals at each stage of study—eg numbers potentially eligible, examined for eligibility, confirmed eligible, included in the study, completing follow-up, and analysed | Page 5 | During the 10-year period, we included 1046 patients with (SRCCE）signet ring cell carcinoma of the esophagus (896 males and 150 females). |
|  |  | (b) Give reasons for non-participation at each stage | N/A |  |
|  |  | (c) Consider use of a flow diagram | N/A |  |
| Descriptive data | 14* | (a) Give characteristics of study participants (eg demographic, clinical, social) and information on exposures and potential confounders | Page 5 | **Table 1** illustrates the distribution of patients’ characteristics in the investigation. Males account for 85.75. The mean (SD) age at diagnosis was 66.8 (11.9) years. 61.3% of included patients are married and white people account for 94.6%. |
|  |  | (b) Indicate number of participants with missing data for each variable of interest | Page 5 | In the Table 1. |
|  |  | (c) *Cohort study*—Summarise follow-up time (eg, average and total amount) | Page 5 | In the Table 1. |
| Outcome data | 15* | *Cohort study*—Report numbers of outcome events or summary measures over time | *Page 8-9* | *Page 8-9* |
|  |  | *Case-control study—*Report numbers in each exposure category, or summary measures of exposure | *N/A* |  |
|  |  | *Cross-sectional study—*Report numbers of outcome events or summary measures | *N/A* |  |
| Main results | 16 | (*a*) Give unadjusted estimates and, if applicable, confounder-adjusted estimates and their precision (eg, 95% confidence interval). Make clear which confounders were adjusted for and why they were included | *Page 8-9* | *In the table2* |
|  |  | (*b*) Report category boundaries when continuous variables were categorized | *Page 8-9* | *In the table2* |
|  |  | (*c*) If relevant, consider translating estimates of relative risk into absolute risk for a meaningful time period | Page 8-9 | **Table 2** demonstrates the results of univariate and multivariate Cox proportional hazards analyses for the mortality-association risk factor in patients with SRCCE. Divorced (HR = 1.467, 95% CI (1.110, 1.938)), separated (HR = 1.332, 95% CI (1.009, 1.759)), tumor size ≥ 5cm (HR = 1.515, 95% CI (1.205, 1.904)), T4 (HR = 1.648, 95% CI (1.275, 2.130)), N1 (HR = 1.485, 95% CI (1.226, 1.798)), M1 (HR = 1.808, 95% CI (1.494, 2.187)) and no treatment (HR = 2.602, 95% CI (1.847, 3.665)) are associated with an increasing risk of mortality, while no clear difference is noted between the prognosis of different surgery and radiotherapy |

Continued on next page

| Other analyses | 17 | Report other analyses done—eg analyses of subgroups and interactions, and sensitivity analyses | N/A |  |
| --- | --- | --- | --- | --- |
| Discussion | | | | |
| Key results | 18 | Summarise key results with reference to study objectives | Page 16 | we found that the 1-, 2- and 5-year DSM were 50.8%, 67.7%, and 78.7%, respectively and the median survival time was 12.0 months, while they are reported poorer in advanced gastric signet ring cell carcinoma derived from anterior investigations **[19]**. |
| Limitations | 19 | Discuss limitations of the study, taking into account sources of potential bias or imprecision. Discuss both direction and magnitude of any potential bias | Page 16-17 | Our analysis was based on the data documented in the SEER database, so we are supposed to recognize some restrictions in our study. First, some variables including comorbidities, surgical margin, the extent of resection, tumor recrudescence and the employment of chemotherapy involved in the management of SRCCE are missing or not recorded in the database. Second, because of the anonymous principle in SEER program, it is impossible for us to contact the patients in order to gain additional information. Finally, it also should not be ignored that with the existence of confounders, the consequences deduced from a retrospective analysis were normally of a lower methodological grade compared with those from randomized controlled trials. Despite the fact that no significant difference is found in these treatments with surgery as the reference, receiving therapy can definitely extend the survival period compared with no treatment.  To sum up, the SEER program database is of significant vantages in spite of these restrictions and provides feasibility for conducting such research on the base of a huge population with rare malignancies. Future explorations ought to be concentrated on the neoplasm biological behaviors and therapeutic efficaciousness of SRCCE. |
| Interpretation | 20 | Give a cautious overall interpretation of results considering objectives, limitations, multiplicity of analyses, results from similar studies, and other relevant evidence | Page 16-17 | Our analysis was based on the data documented in the SEER database, so we are supposed to recognize some restrictions in our study. First, some variables including comorbidities, surgical margin, the extent of resection, tumor recrudescence and the employment of chemotherapy involved in the management of SRCCE are missing or not recorded in the database. Second, because of the anonymous principle in SEER program, it is impossible for us to contact the patients in order to gain additional information. Finally, it also should not be ignored that with the existence of confounders, the consequences deduced from a retrospective analysis were normally of a lower methodological grade compared with those from randomized controlled trials. Despite the fact that no significant difference is found in these treatments with surgery as the reference, receiving therapy can definitely extend the survival period compared with no treatment. |
| Generalisability | 21 | Discuss the generalisability (external validity) of the study results | Page 17 | the SEER program database is of significant vantages in spite of these restrictions and provides feasibility for conducting such research on the base of a huge population with rare malignancies. Future explorations ought to be concentrated on the neoplasm biological behaviors and therapeutic efficaciousness of SRCCE. |
| Other information | |  | | |
| Funding | 22 | Give the source of funding and the role of the funders for the present study and, if applicable, for the original study on which the present article is based | N/A |  |

*Give information separately for cases and controls in case-control studies and, if applicable, for exposed and unexposed groups in cohort and cross-sectional studies.

**Note:** An Explanation and Elaboration article discusses each checklist item and gives methodological background and published examples of transparent reporting. The STROBE checklist is best used in conjunction with this article (freely available on the Web sites of PLoS Medicine at http://www.plosmedicine.org/, Annals of Internal Medicine at http://www.annals.org/, and Epidemiology at http://www.epidem.com/). Information on the STROBE Initiative is available at www.strobe-statement.org.
